# Test your knowledge and understanding

**Published:** 2015

This page is designed to help you test your own understanding of the concepts covered in this issue, and to reflect on what you have learnt. We hope that you will also discuss the questions with your colleagues and other members of the eye care team, perhaps in a journal club. To complete the activities online - and get instant feedback - please visit **www.cehjournal.org**

**Table:** 

**1.**	**Consider diabetes and its link to diabetic retinopathy. Which statement is correct?**	**Select one**
**a**	Diabetes kills fewer people than TB and HIV	□
**b**	The risk of developing type 2 diabetes can be reduced by keeping to a healthy weight, taking regular exercise and minimising sugar intake	□
**c**	Once diabetic retinopathy occurs, the patient will inevitably go blind	□
**d**	If a patient has diabetic retinopathy, they are usually aware of it	□
**2.**	**Consider diabetic retinopathy in individual patients. Which statement is correct?**	**Select one**
**a**	Diabetic retinopathy (DR) affects only people with high blood pressure	□
**b**	If a patient's diabetes and blood sugar levels are well controlled, there is no risk of DR	□
**c**	Laser treatment will improve vision in people with DR	□
**d**	The risk of blindness from DR can be avoided if patients attend hospital appointments as required and accept treatment	□
**3.**	**Consider diabetic eye disease as a public health problem. Which statement is correct?**	**Select one**
**a**	Blindness from diabetic retinopathy can be prevented by a screening programme	□
**b**	Blindness from diabetic retinopathy is mostly linked with patients unable to afford services	□
**c**	The risk of blindness from diabetic retinopathy can be reduced through early detection and treatment	□
**d**	Blindness from diabetic retinopathy occurs mostly in high-income countries	□
**4.**	**Consider the prevention of diabetic eye disease in your district. Which of the following is essential?**	**Select one**
**a**	Good links with other health workers involved in caring for patients with | diabetes (e.g., physicians, diabetes nurses)	□
**b**	A community-based survey to determine the number of people with diabetes in your country	□
**c**	A national diabetic retinopathy screening programme	□
**d**	Enough ophthalmologists to examine every patient with diabetes at least once every year	□

## ANSWERS

1. **Correct answer: b. The risk cannot be abolished by a healthy lifestyle, but it can be greatly reduced.** Diabetes kills more people than TB, malaria, and HIV put together. If diabetic retinopathy is diagnosed and treated promptly, blindness can be prevented in almost all cases. Diabetic retinopathy is usually asymptomatic in its early stages.
2. **Correct answer: d. By regularly attending appointments, DR can be detected early and treated before it causes visual loss. Lifestyle changes can reduce, but not eliminate, the risk of complications.** Anyone with diabetes can develop diabetic eye disease. The duration of diabetes and high blood glucose levels are the most important risk factors. Good control of blood pressure is, however, also very important to reduce the risk of diabetic retinopathy. Blood glucose control reduces the risk of complications, but does not eliminate it. Complications may also be the result of poor control in previous years, even if control is good now. Laser treatment can stabilise the vision, but usually does not improve it. (Laser treatment may be used to prevent the development of proliferative retinopathy or to treat diabetic macular oedema.)
3. **Correct answer: c. Both detection and treatment are needed to reduce the risk of blindness.** Screening alone will not prevent blindness. There must be a referral network to confirm the diagnosis and to deliver effective treatment. Affordability is not the only challenge. Awareness of, access to, and availability of services are also required to prevent blindness. Blindness caused by DR is increasingly common in low-income countries.
4. **Correct answer: a. The support of other health workers is essential.** The number of people with diabetes can be estimated from the global atlas of diabetes. Local, district-level programmes are simpler and cheaper than a national programme. Ophthalmologists may be needed to examine patients with the most advanced retinopathy, but a screening programme may use photographers or other health care workers to examine patients with no or minimal retinopathy.

